# The Maize *Divergent spindle-1* (*dv1*) Gene Encodes a Kinesin-14A Motor Protein Required for Meiotic Spindle Pole Organization

**DOI:** 10.3389/fpls.2016.01277

**Published:** 2016-08-25

**Authors:** David M. Higgins, Natalie J. Nannas, R. Kelly Dawe

**Affiliations:** ^1^Department of Plant Biology, University of GeorgiaAthens, GA, USA; ^2^Department of Genetics, University of GeorgiaAthens, GA, USA

**Keywords:** meiosis, spindle, kinesin-14A, tubulin, maize, mutant

## Abstract

The classic maize mutant *divergent spindle-1* (*dv1*) causes failures in meiotic spindle assembly and a decrease in pollen viability. By analyzing two independent *dv1* alleles we demonstrate that this phenotype is caused by mutations in a member of the kinesin-14A subfamily, a class of C-terminal, minus-end directed microtubule motors. Further analysis demonstrates that defects in early spindle assembly are rare, but that later stages of spindle organization promoting the formation of finely focused spindle poles are strongly dependent on *Dv1*. Anaphase is error-prone in *dv1* lines but not severely so, and the majority of cells show normal chromosome segregation. Live-cell imaging of wild type and mutant plants carrying CFP-tagged β-tubulin confirm that meiosis in *dv1* lines fails primarily at the pole-sharpening phase of spindle assembly. These data indicate that plant kinesin-14A proteins help to enforce bipolarity by focusing spindle poles and that this stage of spindle assembly is not required for transition through the spindle checkpoint but improves the accuracy of chromosome segregation.

## Introduction

The plant cytoskeleton, comprised of actin-based microfilaments and tubulin-based microtubules, is involved in a number of critical cellular processes, including cell elongation, cell wall deposition, and cell division. Microtubules are hollow tube-shaped structures comprised of polymerized dimers of α- and β-tubulin and are polarized into dynamically growing and shrinking plus ends as well as relatively stable minus ends. This polar quality of microtubules is important in a number of their roles in plant cells, including meiosis, the process by which diploid somatic cells undergo reductional division to form haploid gametes. During prophase of meiosis I, microtubules circle the nuclear envelope as the chromosomes inside begin to recombine and condense. As the nuclear envelope breaks down, the minus ends of microtubules organize into the spindle structure while their plus ends attach to kinetochores and position chromosomes in the metaphase plate (reviewed in Howard and Hyman, 2003). Chromosomes are then retracted along the microtubules during anaphase before forming new nuclei in telophase. Organisms have evolved different structures known as microtubule organizing centers (MTOCs) to assist in this process. These include the spindle pole body in budding yeast (reviewed in Kilmartin, 2014), and centrosomes in animals (reviewed in Conduit et al., 2015).

Microtubules in plant cells are not organized by a single structure and are instead nucleated as one of four different arrays throughout the plant cell cycle (reviewed in Lloyd and Chan, 2006; Mcmichael and Bednarek, 2013). During interphase, cortical microtubules are nucleated from multiple, mobile points along the inner surface of the plasma membrane (Chan et al., 2003). Some of these microtubules are capable of slowly depolymerizing at their minus ends while their plus ends elongate in a process known as microtubule treadmilling (Shaw et al., 2003). As the cell prepares to undergo division, microtubules collect around the nucleus. In mitosis, they organize as a ring-shaped structure known as the preprophase band, the location of which predicts the ultimate plate of cell division (Mineyuki, 1999). As the nuclear envelope breaks down at the beginning of cell division, so do the microtubules of the preprophase band which collect in the nuclear space and form a chaotic bipolar array which is then focused and oriented into the spindle (Shamina, 2005). Following anaphase, microtubules nucleate in a flat disc called the phragmoplast between the divided chromosome masses in order to establish the cell membrane and cell wall between the two new daughter cells (Liu et al., 2011). Overall, the cells of flowering plants have unique methods of organizing and nucleating their microtubules compared to other eukaryotes, particularly as they pertain to the spindle structure, the mechanisms of which are still unknown (Zhang and Dawe, 2011).

*Zea mays* (maize) has served as a model for plant cytogenetics research for over a century and a number of meiotic mutants have been identified (Carlson et al., 1988). One such mutant, *divergent spindle-1* (*dv1*), is deficient in meiotic spindle formation in male meiocytes. In wild type meiotic cells, microtubules form a bipolar spindle with organized, focused poles. Cells carrying the mutant *dv1* fail to complete this process with their spindle microtubules remaining unorganized and divergent from one another (Clark, 1940). Chromosomes are retracted along these divergent microtubules during anaphase, causing aberrant chromosome segregation and pollen abortion rates ranging from 56 to 90% in extreme situations (Clark, 1943; Staiger and Cande, 1990). Phenotypically, plants carrying the *dv1* mutation are indistinguishable from wild type siblings, indicating that *dv1* does not cause deleterious effects of mitosis (Staiger and Cande, 1990). Additionally, there is no observed effect on seed set, suggesting the effects of *dv1* are limited to male meiosis and are not present in female meiocytes (Clark, 1940).

Further analysis of *dv1* using immunofluorescence indicated that meiocytes carrying the mutation do not exhibit a microtubule phenotype during prophase, only as the nuclear envelope begins to break down and the spindle starts to organize (Staiger and Cande, 1990). Additional characterization demonstrated a plasticity of the spindle phenotype of *dv1* plants grown under altered light and temperature conditions resulting in a radial spindle phenotype (Shamina et al., 2000). The *dv1* mutation also has effects on the nuclear envelope, resulting in an abnormal breakdown during prometaphase, leaving fragments of the membrane among the chromosome bivalents (Shamina et al., 2000). A recent study supports this view, showing that *dv1* affects localization of the protein SUN2, involved in tethering telomeres to the nuclear envelope (Murphy et al., 2014).

Kinesins are a large superfamily of proteins that are known to be involved in multiple stages of mitosis (reviewed in Hirokawa and Noda, 2008), making them excellent candidates for the gene underlying the *dv1* phenotype. Kinesins were first identified from the extract of squid giant axons by their ability to generate force through binding and releasing microtubules with their highly-conserved motor domain (Vale et al., 1985; Hirokawa et al., 1989). The kinesin superfamily is divided into 14 distinct subfamilies (Lawrence et al., 2004). Each of these subfamilies is distinguished by the unique cargo bound at their tail domains, allowing different kinesins to transport a variety of proteins, vesicles, and organelles throughout the cell (reviewed in Hirokawa et al., 2009). The motor activity of most kinesins is plus end directed, moving only from the minus end of microtubules toward the plus end. The kinesin-14 subfamily is unique in that its members are minus-end directed.

The kinesin-14A subfamily contains multiple examples of genes that are involved in organizing spindle poles from species as diverse as *Saccharomyces cerevisiae* (Meluh and Rose, 1990), *Drosophila melanogaster* (Mcdonald et al., 1990; Walker et al., 1990), *Xenopus laevis* (Walczak et al., 1997), and *Arabidopsis thaliana* (Mitsui et al., 1993). Although their specific phenotypes vary slightly, knockout mutants display errors in cell division, spindle structure and organization of spindle poles (Matthies et al., 1996; Chen et al., 2002). The *Arabidopsis* genome encodes two kinesin-14A genes: Atk5/AtKIN14b (Ambrose and Cyr, 2007), which affects mitotic spindle pole formation, and Atk1/AtKIN14a (Chen et al., 2002), which primarily affects meiotic spindle pole formation and chromosome segregation, similar to maize *dv1* (Quan et al., 2008).

Although the *dv1* mutant was first identified over 75 years ago (Clark, 1940) and has been the subject of several studies since then (Staiger and Cande, 1990; Shamina et al., 2000; Murphy et al., 2014), the gene responsible for its phenotype has never been identified. In this paper, we identify two different members of the kinesin-14A subfamily in maize and demonstrate that the *Dv1* gene encodes one of these proteins through sequencing, quantification of transcripts, and allelism tests of two alleles we identify as *dv1-1* and *dv1-IG*. In addition, we further characterize the effects of *dv1* by quantifying spindle shape in the *dv1* mutant using immunolocalization, examining the link between errors in meiosis and pollen viability, and documenting the effects of *dv1* on spindle assembly and chromosome segregation *in vivo* through live cell imaging. Overall, we find that *Dv1* is not required for the formation of bipolar spindles but is specifically required for focusing the spindle pole to a fine point. These data suggest that in plant cells, as in animal oocytes lacking centrosomes (Matthies et al., 1996; Walczak et al., 1998), kinesin-14A proteins serve the primary function of organizing spindle poles.

## Materials and methods

### Maize tissue and DNA extraction

Maize stocks for *dv1-1* and *dv-IG* were received from the Maize Genetics Cooperation Stock Center (University of Illinois). The inbred A619 used in the EMS mutagenesis that led to the identification *dv1-IG* was received from Jay Hollick (Ohio State University). Seeds carrying the CFP-tubulin transgene were received from Anne Sylvester (University of Wyoming). DNA extractions were carried out on leaf tissue using a CTAB protocol with ethanol precipitation (Clarke, 2009).

### DNA sequencing, *dv1* allele genotyping, and qRT-PCR

Genomic DNA was amplified using the Phusion High-Fidelity PCR Kit (New England Biolabs, Ipswich, MA) with the primers outlined in Table S1. PCR products were purified for sequencing using the QIAquick PCR Purification Kit (QIAGEN, Germantown, MD). Sanger sequencing was completed at the Georgia Genomics Facility (Athens, GA). Reads were aligned to the B73v3 maize reference genome using the software Geneious (v8.0, Biomatters Ltd., Auckland, New Zealand).

For subsequent studies, *dv1-1* and *dv1-IG* were differentiated from wild type alleles using PCR and restriction digest. Amplification of maize genomic DNA using the *dv1-1* genotyping primers (Table S1) produces a PCR product of 235 bp. When this product is digested with the restriction endonuclease MseI (New England Biolabs), the wild type copy remains uncut while *dv1-1* is cleaved into two pieces of sizes 152 and 83 bp. Likewise, *dv1-IG* genotyping primers (Table S1) produce a product of 533 bp. When digested with the enzyme NsiI (New England Biolabs), the *dv1-IG* allele remains uncut while the wild type allele will be cleaved into two pieces of sizes 272 and 261 bp.

For quantification of *dv1* transcripts, RNA was extracted from meiotic anthers using the RNeasy Plant Mini Kit (QIAGEN, Germantown, MD) from which cDNA was generated using the SuperScript III First-Strand Synthesis System for RT-PCR (Thermo Fisher Scientific, Waltham, MA). cDNA was quantified using a Qubit 3.0 Fluorometer (Thermo Fisher Scientific), and equal amounts of template were used for a qRT-PCR reaction with Power SYBR Green PCR Master Mix (Thermo Fisher Scientific). Primers used for *dv1* quantification are given in Table S1 while primers for reference gene Membrane protein PB1A10.07c (MEP) were previously published (Manoli et al., 2012). Quantification of transcripts was carried out using the $2^{-\Delta \Delta {\text{C}}_{\text{T}}}$ method (Livak and Schmittgen, 2001).

### Meiocyte immunolocalization

Immunolocalization of maize meiocytes was carried out using an altered version of the protocol by Staiger and Cande (1990). Whole anthers containing meiocytes at metaphase were dissected from florets and fixed in 4% paraformaldehyde in PHEMS buffer for 60 min. Fixed anthers were washed in PBS and meiocytes were extruded onto coverslips coated in poly-L-lysine. Coverslips were spun in a swinging bucket centrifuge at 100 g for 1 min to affix meiocytes. Meiocytes were incubated for 1 h in a permeabilization solution (1% Triton X-100 in PBS with 1 mM EDTA) before being blocked in 10% goat serum for 90 min. Meiocytes were incubated with a monoclonal antibody against sea urchin α-tubulin (Asai et al., 1982) at 37°C overnight. Coverslips were again blocked with 10% goat serum, followed by incubation with Rhodamine-conjugated AffiniPure Goat Anti-Mouse IgG (H+L) secondary antibodies (Jackson ImmunoResearch Inc., West Grove, PA) for 150 min. Washed coverslips were mounted in ProLong Gold with DAPI (Thermo Fisher Scientific). Slides were viewed on a Zeiss Axio Imager. M1 fluorescence microscope with a 63x Plan-APO Chromat oil objective. Images were collected using the Slidebook software package (Intelligent Imaging Innovations, Denver, CO) at exposure times ranging from 0.1 to 1 s. Spindle measurements were collected using the Line tool in the Slidebook software. Statistical significance was determined through an ANOVA using the R package “agricolae” (de Mendiburu, 2015).

Immunolocalization for scoring the multinucleate daughter cells was conducted in the same manner as described above with a primary antibody specific to CENP-C (Dawe et al., 1999) and fluorescein-conjugated AffiniPure Goat Anti-Mouse IgG (H+L) secondary antibodies (Jackson ImmunoResearch Inc.).

### Pollen viability

Fresh pollen was collected from dehiscent tassels and stained using a modified version of Alexander's stain (Alexander, 1969; Peterson et al., 2010). Slides were viewed on a Zeiss Axiophot light microscope using a 10x Plan-NEOFLUAR objective lens. A total of 500 pollen grains from each plant were scored for viability based on color. Statistical significance was determined through an ANOVA using the R package “agricolae” (de Mendiburu, 2015).

### Live meiotic imaging and analysis

Cells in metaphase I were extruded from immature maize tassels as previously described (Yu et al., 1997) into a meiocyte-specific live cell imaging medium (De La Peña, 1986; Yu et al., 1997) that contained a final concentration of 2 μM SYTO12 Green DNA dye (Invitrogen Molecular Probes, Grand Island, NY). Coverslips were sealed and slides imaged using the same microscope, objective and software used for immunolocalization. Images were collected at 5-min intervals in 3-dimensions using a 20 μm Z range and step size of 1 μm. In order to minimize photo-bleaching, exposure times were kept brief (30 ms for SYTO12 and 50 ms for CFP) and binning was increased (2 × 2). Chromosome offset and anaphase movements were measured using Slidebook; the three-dimensional coordinates of the center of spindle and chromosome masses were extracted using the object statistics function. Chromosome offset was measured by calculating the distance between the chromosome and spindle coordinates. Anaphase distances were calculated as the three-dimensional distance between the chromosomes at different time points, and anaphase rate was calculated as this distance divided by time.

## Results

### Identification of mutations associated with the *dv1* phenotype

**Figure 1:**
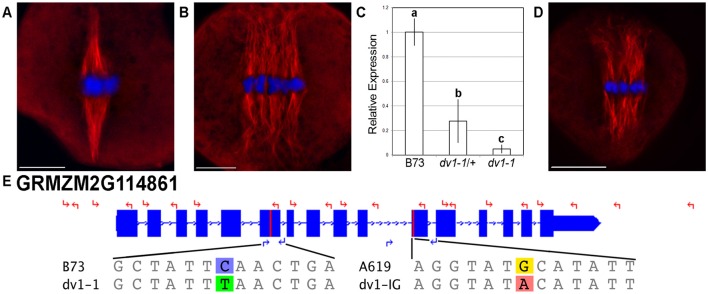
**Two alleles of ***dv1*** show a divergent spindle phenotype**. Meiocytes were stained using immunofluorescence with a primary antibody specific to α-tubulin. Scale bars for each image represent 10 μm. **(A)** Wild type spindle showing highly focused spindle poles; **(B)** *dv1-1/dv1-1* spindle showing splayed, divergent poles; **(C)** Expression of the *dv1-1* allele of ZmKin6 adjusted to the B73 wild type allele. Error bars represent 95% confidence intervals, groups of significant difference are designated with lowercase letters; **(D)** *dv1-1/dv1-IG* heteroallelic mutant is similar to the *dv1-1* homozygote; **(E)** Gene model of *ZmKin6* highlighting the location of the two *dv1* alleles, the *dv1-1* stop codon in the sixth exon and the *dv1-IG* transversion in the motor domain. Coordinates along chromosome 2 are shown above. The locations of sequencing primers are shown with red arrows while the locations of genotyping primers are shown with blue arrows. The reference and mutant sequences of each allele are shown below the gene diagram.

Based on the observation that the *dv1* phenotype in maize (Figures 1A,B) is similar to mutations in members of the kinesin-14A subfamily in other species (Matthies et al., 1996; Chen et al., 2002), we hypothesized that the gene underlying the *dv1* phenotype may be a member of this subfamily as well. The sequences of genes of this subclass were collected from different species, including the *Arabidopsis* genes AtKIN14a (Chen et al., 2002), and AtKIN14b (Ambrose et al., 2005), the *Drosophila melanogaster* gene NCD (Mcdonald et al., 1990) and the *Xenopus laevis* gene XCTK2 (Walczak et al., 1997). In addition, we extracted a complete list of maize genes containing the kinesin motor domain from the maize B73 reference genome (Schnable et al., 2009). The coding sequences (CDS) of these genes were aligned using the MUSCLE algorithm (Edgar, 2004) and assembled into a gene tree using RAxML (Stamatakis, 2006) in order to identify members of the kinesin-14A subfamily in maize. The resulting tree identified two maize genes that cluster with the kinesin-14As of other species (Figure S1). These two genes are GRMZM2G114861 and GRMZM2G436981, previously annotated as *ZmKin6* and *ZmKin11*, respectively (Lawrence et al., 2002). Transcriptome data on these two genes indicates that the expression of *ZmKin6* in meiotic anthers is 5.18-fold higher than *ZmKin11* (Sekhon et al., 2011, accessed via Maize eFP Browser). Given this large difference in expression in the tissue that shows the *dv1* phenotype, we focused our efforts on sequencing and characterizing *ZmKin6* in the *dv1* background.

We generated primer pairs (Table S1) for Sanger sequencing across the exons of *ZmKin6* in a maize line that contained the reference *dv1* allele identified by Clark (1940), which we refer to as *dv1-1*. We identified two single nucleotide polymorphisms (SNPs) in the *dv1-1* allele of *ZmKin6* (Data Sheet S1). One SNP produces a premature termination codon (PTC) in the sixth exon of the predicted peptide (Figure 1E). As this mutation is upstream of the kinesin motor domain, any mRNA that is produced is likely to be nonfunctional. The second SNP causes a transversion in the eleventh exon, downstream of the PTC and likely to be of no consequence. We also assayed mRNA from the mutant to determine whether nonsense mediated decay, a mechanism that eliminates transcripts carrying deleterious alleles, causes a reduction in the level of ZmKin6 transcript. We outcrossed *dv1-1* to the maize inbred B73 and self-crossed to create a segregating F2 population with *dv1-1*/*dv1-1, dv1-1*/+, and wild type genotypes. Quantitative RT-PCR on meiotic anthers from each of these indicates that expression of *dv1* in the *dv1-1/*+ genotype is approximately 30% of wild type while *dv1-1/dv1-1* is 5% of wild type (Figure 1C).

We also received from Inna Golubovskaya (University of California, Berkeley, retired) an independently-generated mutant line that shows a similar phenotype as *dv1* (Cande and Freeling, 2011). We renamed this previously unpublished mutant, originally referred to as ‘divergent EMS new’ *dv1-IG* in her honor. The same primers used to identify the *dv1-1* allele were used to sequence across the *ZmKin6* gene in *dv1-IG* and the A619 inbred from which *dv1-IG* was derived. We identified eight SNPs that differentiated the *dv1-IG* allele from the B73 reference (Data Sheet S1). Seven of these were silent mutations found in both *dv1-IG* and the progenitor A619. However, the eighth is unique to the *dv1-IG* allele and results in a transition of the 494th amino acid from a cysteine to a tyrosine residue (Figure 1E). This mutation is in the highly conserved kinesin motor domain, within five amino acid residues of the ATP-binding pocket (Kull et al., 1996; Sablin et al., 1996). We used the software tool PROVEAN to predict the effects of this mutation based on levels of conservation between homologous genes in other species (Choi et al., 2012; Choi and Chan, 2015). PROVEAN generates an alignment of homologous genes and returns a value between +4 and −13, representing a range of predictions for a given mutation to be somewhere between neutral and highly deleterious, respectively (Choi et al., 2012). Based on the high levels conservation of this site across plant species, the PROVEAN software predicts that the *dv1-IG* allele of *ZmKin6* would be deleterious with a score of −10.789 (Figure S2). We conducted an allelism test by crossing a line homozygous for the *dv1-1* allele with a line heterozygous for the *dv1-IG* allele. The heteroallelic progeny (*dv1-1/dv1-IG*) showed a clearly divergent spindle phenotype (Figure 1D, *n* = 6). Taken together, these data indicate that *ZmKin6* is the *Dv1* gene.

### The *dv1-1* allele affects meiotic spindle shape and length as a homozygote and heterozygote

**Figure 2:**
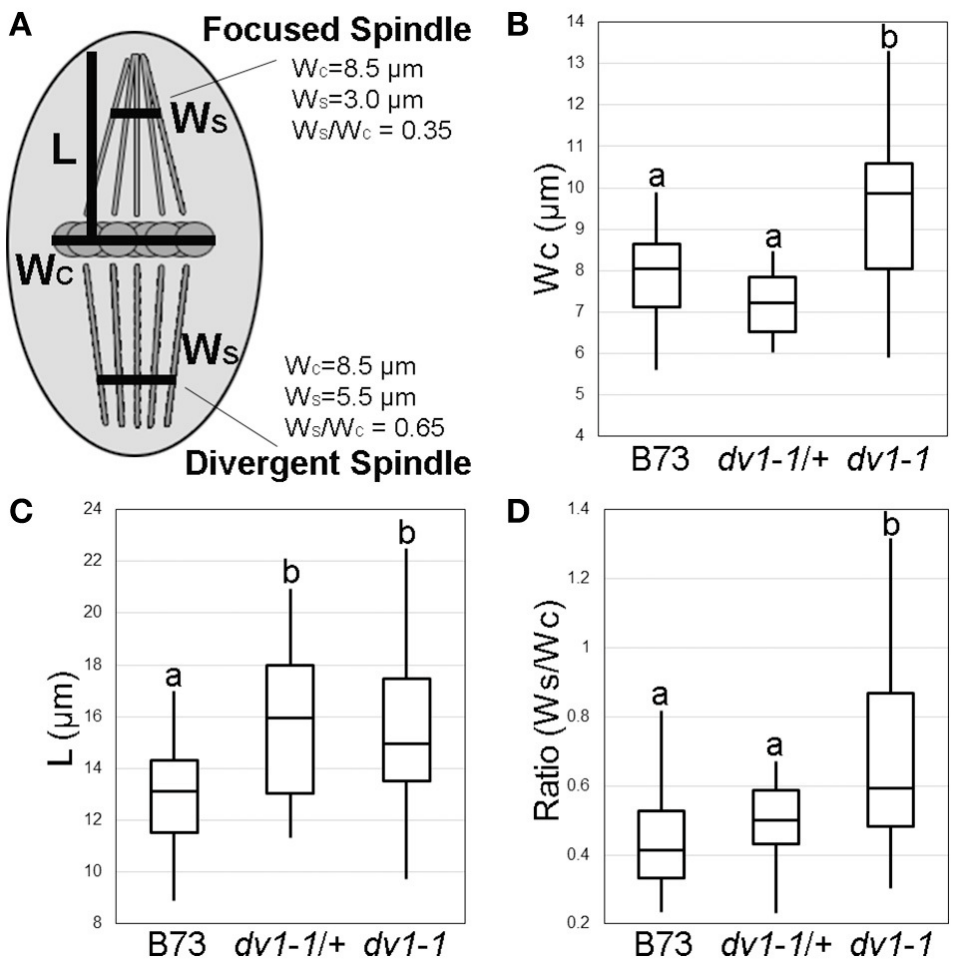
**Quantification of the ***dv1-1*** phenotype on spindle shape and pollen viability**. Spindles were visualized using immunofluorescence as shown in Figure 1. Measurements were taken using the Slidebook 6 digital microscopy software package (Intelligent Imaging Innovations, Denver, CO). Lowercase letters in B, C, and D represent groups of significant difference as determined using ANOVA at α = 0.05. **(A)** Schematic of measurements taken, including width of the metaphase plate (W_*C*_), length of the half-spindle from metaphase plate to pole (L), and width of the spindle at 75% of length L (W_*S*_). The ratio of W_S_/W_C_ can be used to quantify spindle shape as either focused (shown in the top half-spindle) or divergent (bottom half-spindle); **(B)** Width of the metaphase plate (μm) is significantly higher in plants homozygous for *dv1-1* than either wild type or heterozygous plants; **(C)** Length of the half-spindle (μm) is significantly larger than wild type in both *dv1-1* homozygotes and heterozygotes; **(D)** The ratio of spindle width at the metaphase plate to spindle width near the poles, a proxy for spindle shape and degree of pole focus, is significantly higher in *dv1-1* plants while heterozygotes displayed an intermediate phenotype.

The segregating F2 population described above was also used for a more thorough imaging analysis of homozygous mutant (*dv1-1*/*dv1-1*), heterozygous (*dv1-1*/+), and wild type (B73) siblings. Spindle shape was quantified by measuring the width of the metaphase plate (W_C_), the length of the half-spindle (L), and the width of the spindle at 75% of the length of the half-spindle (W_S_) (Figure 2A). The metaphase plate was found to be significantly wider in *dv1-1* mutants than wild type or heterozygous siblings (Figure 2B, α = 0.05). Half-spindle length was determined to be significantly longer in plants carrying *dv1-1* as both a heterozygote and a homozygote (Figure 2C, α = 0.05). Due to the differences in W_C_, we opted to correct the comparison of W_S_ measurements by the width of the chromosomes. The resulting ratio of spindle width to metaphase plate width (W_S_/W_C_) represents how tightly focused the spindle is: a value closer to 1 indicates that the spindle microtubules have not converged while a value closer to 0 indicates a very narrow, tightly organized spindle (Figure 2A). When spindle width was measured at the distance of 75% along its half-length, spindle shape was significantly more diverged in *dv1-1* mutants than in either wild type or heterozygous siblings. However, we also observed that heterozygotes displayed an intermediate phenotype suggestive of partial dominance (Figure 2D). Our data show that the mode of inheritance for the effects of *dv1-1* varies for different phenotypes (Table 1).

**Table 1:** **Summary of the modes of inheritance on different ***dv1-1*** phenotypes**.

**Phenotype**	**Mode of Inheritance**
Spindle length (L)	DOMINANT
Metaphase plate width (Wc)	RECESSIVE
Spindle shape (Ws/Wc)	RECESSIVE
Pollen viability	SEMI-DOMINANT

### Errors in chromosome segregation and pollen viability

**Figure 3:**
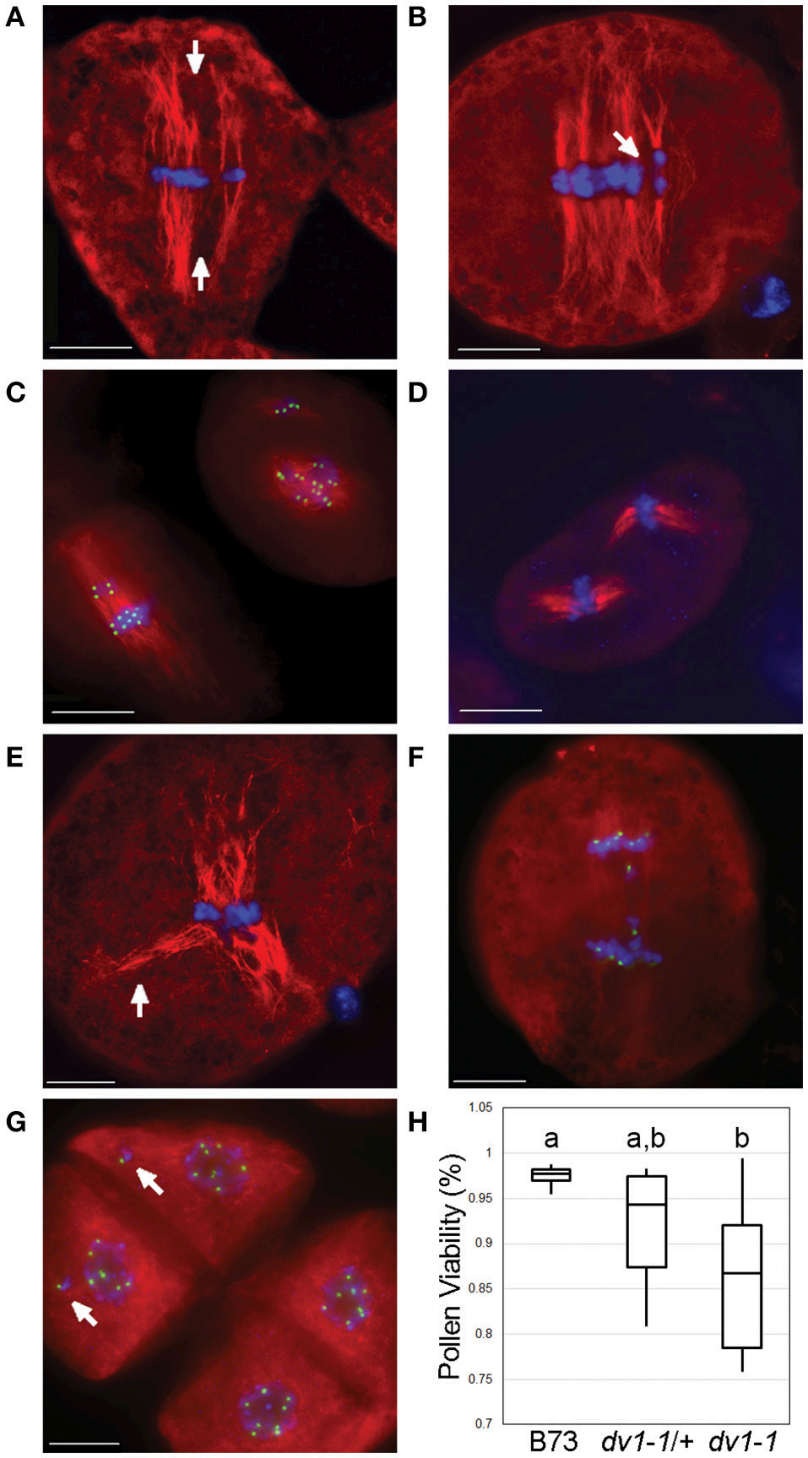
**Rarely-observed severe effects of ***dv1-1*** include multiple spindles and multinucleate daughter cells**. Meiocytes were stained using immunofluorescence with a primary antibody specific to α-tubulin shown in red and CENP-C shown in green. DNA (DAPI) is shown in blue. Scale bars for each image represent 10 μm. **(A)** Multi-spindle *dv1-1* cell with metaphase alignment errors. Poor congression of chromosomes along the metaphase plate in a *dv1-1* cell with parallel spindles in metaphase separated by space (arrows); **(B)** Early anaphase of a similar *dv1-1* cell with parallel spindles and chromosomes separated by space (arrow); **(C)** Cells with severe errors in spindle assembly. Cell on the left appears to be a fusion of a small and a large spindle in the process of correction. Cell on the right shows two distinct spindles, the larger of which appears as a chaotic array of early prometaphase; **(D)** Two separate spindles of approximately the same size in a *dv1-IG* cell; **(E)** Tri-polar spindle of a *dv1-1* cell at metaphase showing separated microtubules (arrow) with congressed chromosomes; **(F)** Lagging chromosomes during anaphase in a *dv1-1* cell; **(G)** Tetrad-stage cells showing examples of mininuclei (arrows), isolated chromosomes not a part of the nucleus; **(H)** Pollen shows a decreased viability in both *dv1-1* homozygotes and heterozygotes. Groups of statistical significance are designated with lowercase letters. The heterozygous phenotype was not statistically significant from either wild type or *dv1-1* homozygous. (*n* = 7–10 plants per genotype, 500 pollen grains per plant; α = 0.05).

Prior data suggest that a small percentage of *dv1* cells contain severely perturbed spindles (Staiger and Cande, 1990). We also observed similar phenotypes. For instance, some spindles appear to be divided into separate, smaller spindles parallel to one another (Figures 3A,B). We also observed prometaphase cells with entirely separate spindles oriented in different directions in both the *dv1-1* (Figure 3C) and *dv1-IG* homozygotes (Figure 3D). Multiple mini-spindles within a single cell have been reported in meiosis II of the *Arabidopsis AtKin14A* mutant, similar to what we have observed here (Chen et al., 2002). One particularly abnormal spindle showed the opposite phenotype, with congressed chromosomes but a very distinct, tri-polar spindle (Figure 3E). These extreme phenotypes were only observed in homozygous mutants, suggesting the heterozygous genotype is not sufficient to produce such aberrant spindles.

Although such severe spindle defects seem likely to lead to errors in chromosome segregation, we rarely observed lagging chromosomes at anaphase (Figure 3F). One way to assay chromosome loss during meiosis is to score at the tetrad stage where lost chromosomes are visible as “mininuclei” that are separated from primary telophase nuclei (Figure 3G). We therefore analyzed tetrad-stage cells derived from the same F2 siblings segregating for *dv1-1*. Kinetochores were visualized using an antibody to Centromere Protein C (CENP-C) as a means to distinguish chromosomes from one another (Dawe et al., 1999). Less than one percent of *dv1-1* cells at the tetrad stage (3 of 434 counted) showed a multinucleate phenotype, demonstrating that while meiotic spindle shape is clearly aberrant in *dv1-1* plants, the majority of cells segregate chromosomes correctly.

The *dv1* mutant is known to affect pollen viability (Clark, 1940). This phenotype could be caused by loss of chromosomes from aberrant meioses, errors in the mitotic cell divisions that precede pollen formation, or some other cause. It has been shown that mature pollen collected from *dv1-1* plants often contain fewer nuclei than expected (one or two nuclei instead of three), and that pollen with an abnormal number of nuclei are less likely to germinate (Clark, 1943). We stained pollen from the same F2 population using a modified version of Alexander's stain that differentiates viable pollen from empty exine (Peterson et al., 2010). Mean pollen viability was significantly lower in *dv1-1* mutants, showing a reduction from 97.7% in wild type to 86.8% in mutant plants (Figure 3H, α = 0.05). The viability of pollen in the *dv1-1/*+ heterozygote was an intermediate value of 94.3% that was not significantly different from either wild type or *dv1- 1* mutants (Figure 3H, α = 0.05). These results suggest that the effects of *dv1-1* on pollen viability are not necessarily an outcome of spindle defects in meiosis and may be caused by errors in the mitotic divisions during gametophyte development.

### Live cell imaging of *dv1* implicates specific roles for Kinesin-14As in spindle assembly

The static images provided by fixed specimens have limited value when studying a dynamic process, such as spindle assembly. To observe the *dv1-1* phenotype in live cells, we crossed *dv1-1* into a maize line containing a version of β-tubulin tagged with cyan fluorescent protein (Mohanty et al., 2009). Meiocytes carrying the CFP-tubulin transgene were extruded from anthers into a growth medium containing SYTO12, a fluorophore that stains DNA in live cells (Yu et al., 1997), allowing for two-color imaging of spindle microtubules and chromosomes. Viable cells in prometaphase and metaphase are difficult to find and rarely continue through anaphase under observation; however, we were able to image several live meiocytes from wild type, *dv1-1/*+, and *dv1-1* plants.

**Figure 4:**
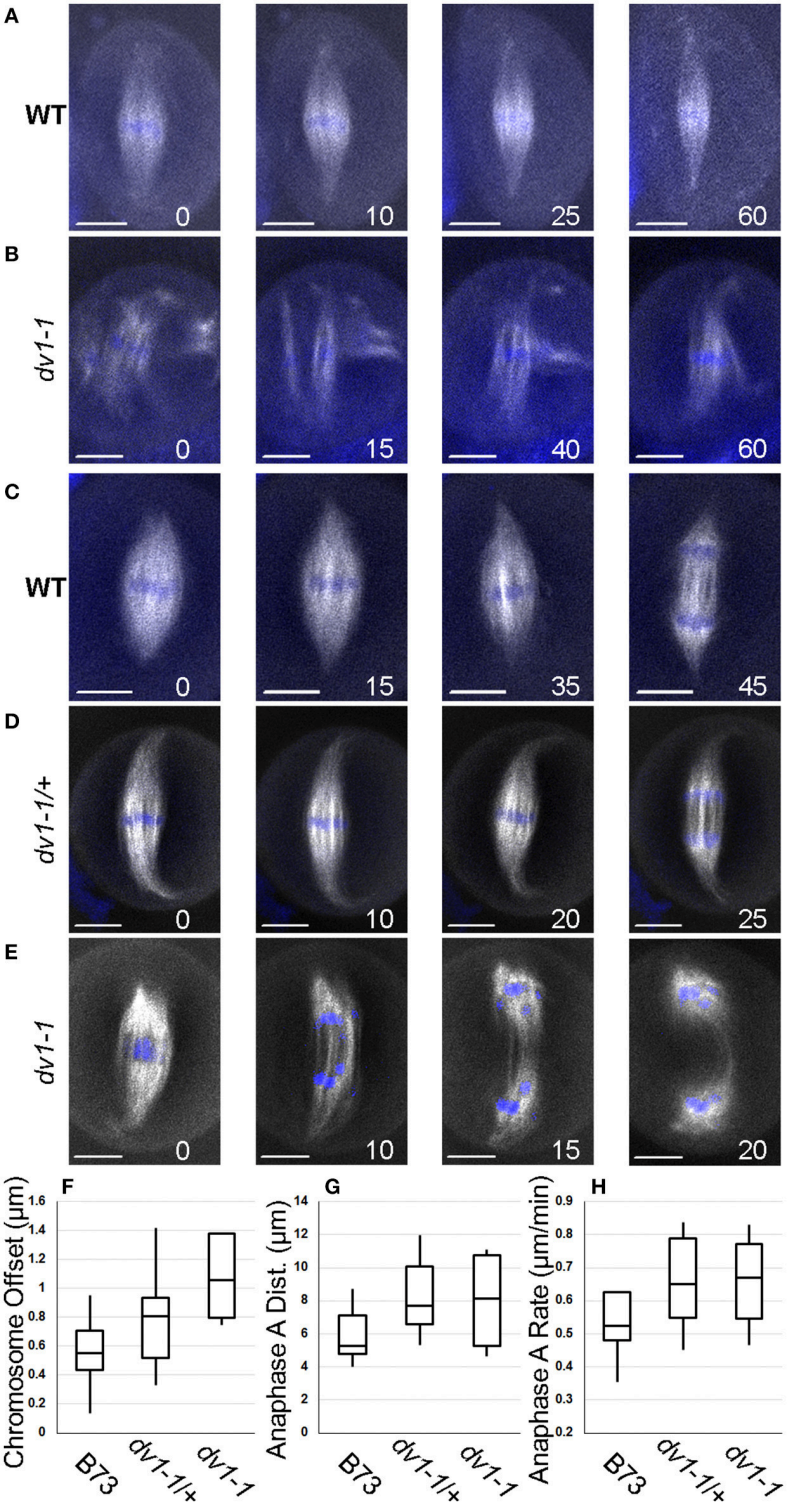
**Live cell imaging demonstrates a role for ***Dv1*** in prometaphase and metaphase**. Images captured from a line carrying a β-tubulin transgene tagged with a cyan fluorescent protein (shown in white) and incubated with SYTO12 which stains chromosomes (shown in blue). Images presented are sequential frames from a single movie with minutes since the original capture shown in the lower right of each image. Scale bars for each image represent 10 μm. Movies of all cells can be found in Movie S1. **(A)** Chromosomes in a wild type cell begin loosely collected in a metaphase plate and then compress as the spindle narrows throughout metaphase; **(B)** Chromosomes in a *dv1-1* cell are highly unorganized in prometaphase with three separate mini-spindles around different chromosome groups which are then brought together; **(C)** The spindle poles of a wild type cell begin rounded, then sharpen and elongate before the cell enters anaphase; **(D)** The spindle pole of a heterozygous cell shows highly focused spindles which curl along the edge of the cell as it enters anaphase; **(E)** The spindle pole of a *dv1-1* cell is highly unorganized in metaphase and anaphase chromosome movement is uneven with several lagging chromosomes; **(F)** Measurement of chromosome offset, the distance between the center of the chromosome mass and the spindle appears to be larger in the *dv1-1* homozygote than other genotypes; **(G)** Distance of chromosomes moved in anaphase A appears to be larger in the *dv1-1* heterozygotes and homozygotes; **(H)** Rate of chromosome movement in anaphase A is not significantly different between the three genotypes.

In wild type cells, prometaphase chromosomes begin in close proximity to one another and only mild adjustments are needed to align them in the metaphase plate (Figure 4A). In contrast, the chromosomes of a *dv1-1* cell begin much further apart and require more dramatic movement to form a metaphase plate (Figure 4B). Separate microtubule spindles attach to distinct groups of chromosomes and then fold and connect with one another as the chromosomes begin to congress (Figure 4B). These data corroborate the findings from our immunolocalization imaging showing multiple smaller spindles forming in *dv1-1* mutants (Figures 3C,D).

Additional live cell imaging shows chromosome and spindle dynamics in metaphase and anaphase. As a wild type cell prepares to enter anaphase, the poles of the metaphase spindle focus from a rounded tip to a pointed tip before the chromosomes divide (Figure 4C). A heterozygous *dv1-1/*+ cell shows a similar phenotype with the spindle tips curling along the edge of the cell (Figure 4D). A third series of images from a *dv1-1* homozygote shows that the spindle pole never becomes fully focused before anaphase begins. (Figure 4E). While chromosome segregation toward the poles is uniform in both the wild type and *dv1-1/*+ cells, lagging chromosomes were observed in the *dv1-1* homozygote, a phenotype seen in fixed cells as well (Figure 3F). A full time series of the data shown in Figure 4 can be seen in supplemental Movie S1 while additional movie data captured but not shown here can be found in Movie S2.

To quantify the observed effects of *dv1-1* on spindle morphogenesis, we measured three separate phenotypes: chromosome congression, anaphase segregation distance and rate of chromosome movement. Due to difficulty of capturing living meiocytes undergoing division, the sample size is low and not amenable to statistical analysis (*n* = 4 to 12 per genotype). We defined chromosome offset as the distance between the center of the spindle and the center of the mass of chromosomes at metaphase. This value assesses how well the chromosomes are congressed and aligned in the middle of the spindle. The trends from our data suggest that *dv1-1* negatively affects the cell's ability to organize and collect chromosomes on the spindle (Figure 4F). Anaphase A is the movement of the chromosomes from the metaphase plate toward the poles, and is the result of the retraction of chromosomes along the kinetochore microtubules. The trends in our data show that chromosomes move slightly farther in both the *dv1-1/*+ and *dv1-1* genotypes (Figure 4G). The rate of chromosome movement in anaphase A is very uniform across all three genotypes (Figure 4H), suggesting that the difference in anaphase A distance is due to movement over a longer time rather than a faster movement. The genotypes with farther moving chromosomes are the same as those with longer spindles *(dv1-1 and dv1-1/*+, Figure 2C) and the possible relationship between these phenotypes is discussed below.

## Discussion

We demonstrate that the maize *Dv1* locus encodes the kinesin-14A gene GRMZM2G114861, previously described as *ZmKin6* (Lawrence et al., 2002). The reference allele *dv1-1* contains a stop codon in the middle of the gene that results nonsense mediated decay of the transcript while a second allele *dv1-IG* contains a deleterious mutation in the conserved motor domain. Both alleles affect spindle shape (Figure 2D) and pollen viability (Figure 3H). The fact that kinesin-14A mutants in other species have very similar phenotypes to *dv1* (Matthies et al., 1996; Chen et al., 2002; Ambrose and Cyr, 2007) provides further evidence supporting this conclusion.

Our quantitative analysis of spindle shape suggests that *dv1* affects both the width and length of the half-spindle, with the effect on half-spindle length being genetically dominant (Table 1). The large decrease in *Dv1* expression observed in heterozygous lines (Figure 1C) suggests that the semi-dominant effects we observed may be due to haploinsufficiency. We also found that pollen viability is reduced in both *dv1* homozygotes and heterozygotes (Table 1) although the effects on pollen viability were not as severe as those previously described by either Clark (as low as 10% viable, 1943) or Staiger and Cande (approximately 44% viable, 1990). Shamina et al. (2000) demonstrated a dramatic effect on spindle shape in *dv1* plants grown under altered light and temperature conditions, suggesting that the *dv1* phenotype may depend heavily on genetic background and environmental conditions.

Our data support the prevailing view that the major role of C-terminal kinesins, such as DV1 in spindle assembly is to gather microtubules and focus the spindle poles (Sharp et al., 2000). The model presented by Hepperla et al. (2014) suggests that kinesin-14 motors are capable of cross-linking antiparallel microtubules from the two different spindle poles, allowing them to tighten and slide along one another. Measurements of wild type and *dv1-1* spindles indicate that mutant spindles are wider at both the metaphase plate (Figure 2B) and 75% along the length of the half-spindle (Figure 2D), supporting a role for kinesin-14As in pulling microtubules together. Our measurements of half-spindle length indicate *dv1-1* mutants have longer spindles than wild type (Figure 2C), contrary to findings of other kinesin-14A studies in mitotic cells of humans (Cai et al., 2009) and yeast (Troxell et al., 2001). The *Arabidopsis* homolog *AtKin14B* showed no effect on spindle length at metaphase in mitotic tissues (Ambrose and Cyr, 2007). Our finding of increased spindle length could be the result of the lack of a canonical MTOC in plant cells, a unique effect of meiosis, or some factor of both. Overall, microtubules in wild type cells and *dv1-1* cells appear similar early in metaphase, where spindles are only loosely focused. It is only later in organization of the spindle structure that the phenotype becomes obvious, as wild type cells proceed to form tightly focused poles, but *dv1-1* spindles remain in a loosely focused state.

While DV1 is not strictly required for the separation of chromosomes, we observed negative effects in anaphase, such as lagging chromosomes in the *dv1-1* mutant (Figures 3F, 4E). Homozygous *dv1-1* plants nevertheless produced ample functional pollen (Figure 3H) and assays for micronuclei (Figure 3G) showed that errors in chromosome segregation are rare. One explanation for the limited phenotypic consequences of *dv1* may be genetic redundancy, as the second kinesin-14A gene in maize GRMZM2G436981 (Lawrence et al., 2002) is expressed at a low level in anthers (Sekhon et al., 2011) and may be involved in spindle morphogenesis as well.

While the major effects of *dv1* on microtubule organization are not visible until late in the organization of the meiotic spindle, *dv1-1* has been shown to have effects on nuclear envelope prior to its breakdown at the onset of metaphase. The localization of ZmSUN2, a protein that functions as a part of complex that bridges chromatin and the cytoskeleton during meiotic prophase is aberrant in *dv1-1* mutants (Murphy et al., 2014), and fragments of the nuclear envelope remain among the chromosomes as late as metaphase (Shamina et al., 2000). A survey of male meiosis in several species of land plants indicates that the male meiotic spindle is derived from a “chaotic array” of bipolar microtubules which crash into the nuclear space following envelope breakdown (Shamina, 2005). Our movies of wild type meiocytes match this observation with a single large spindle quickly forming and focusing (Figures 4A,C). In *dv1-1* cells, we observed several small spindles independently forming around separate groups of chromosomes which were then pulled together (Figures 3A–D). Perhaps poor collection of the chromosome bivalents in the nucleus during prophase, a result of mislocalization of SUN2 in the absence of DV1 (Murphy et al., 2014), causes chromosomes to be more dispersed following nuclear envelope breakdown. Live cell imaging shows that meiocytes are capable of at least partially correcting this error (Figure 4B), but nuclear envelope defects could be partially responsible for the some of the spindle phenotypes observed in *dv1* meiocytes, such as the increased size of the metaphase plate (Figure 2B) and decreased chromosome alignment on the spindle (Figure 4F). Future studies on the physical interactions between DV1 and other proteins at the surface of the nucleus are needed to explore this potential mechanism.

In animals where centrosomes facilitate spindle formation, the minus-end directed motor dynein has a key role is focusing spindle poles (Sharp et al., 2000). However, the oocytes of many animals are anastral, lacking centrosomes, and form by a mechanism whereby the kinesin superfamily of proteins has a more prominent role in organizing poles (Walczak et al., 1998). Higher plants lack dynein (Lawrence et al., 2001) and their spindles are presumed to form by a mechanism similar to animal anastral spindles, thus relying more heavily on the activity of kinesins (Bannigan et al., 2008; Zhang and Dawe, 2011). Our data on *dv1* provides direct supporting evidence for this model of kinesin-driven spindle assembly. While still able to form a metaphase plate and bundle microtubules, reduction of kinesin-14A inhibits the formation of the spindle poles, causing a mild reduction in the accuracy of chromosome segregation and viability of resulting daughter cells.

## Author contributions

DH contributed to the design of the experiments, collection of data, analysis, and writing of the manuscript. NN contributed to the collection of data, analysis, and edited the manuscript. RD contributed to the design of the experiments, analysis of data, writing and editing of the manuscript, and funding.

### Conflict of interest statement

The authors declare that the research was conducted in the absence of any commercial or financial relationships that could be construed as a potential conflict of interest.

## Supplementary material

Figure S1 **Alignment of maize kinesins reveals two members of the kinesin-14A subfamily**. Coding sequences were aligned using the MUSCLE algorithm. All genes shown are from the *Zea mays* B73 reference genome unless a different species name is given. Two maize genes appear with other members of the kinesin-14A subfamily (highlighted in red) and were identified as candidates for *dv1*. The figure was generated using the software Geneious (v8.0).

Figure S2 **PROVEAN output indicates the residue at which the *dv1-IG* mutation occurs is highly conserved**. The PROVEAN software identified 75 GenBank entries that show homology to the *Dv1* sequence. GenBank IDs are listed for each entry along with their species and given annotation. A portion of the PROVEAN alignment is shown at the right. Amino acid sequences for the B73 reference and *dv1-IG* allele are listed at the top. This cysteine in the middle of the alignment (residue 494) is conserved across the sample of plant, algae, and animal sequences. However, the *dv1-IG* allele codes for a tyrosine at this position. The figure was generated using the software Geneious (v8.0).

Table S1 **Complete list of DNA primers used in this study**.

Movie S1 **Meiotic spindle assembly and cell division in wild type and *dv1-1* cells**. Five separate meiotic division events are shown in succession corresponding to Figures 4A–E. Each frame represents 5 min in real time. Scale bars shown are 10 μm. Microtubules tagged with CFP-tubulin are shown in blue while SYTO12 staining DNA is shown in green.

Movie S2 **Additional movies of meiotic spindle assembly and cell division**. Eight separate meiotic division events are shown in succession. Each frame represents 5 min in real time, scale bars shown are 10 μm. Microtubules are shown in white and chromosomes are shown in blue.

Data Sheet S1 **Sequence of four alleles of Dv1 (B73, *dv1-1*, A619, *dv1-IG*) in FASTA format**.
