# Supplementary figures and images for: The Maize Divergent spindle-1 (dv1) Gene Encodes a Kinesin-14A Motor Protein Required for Meiotic Spindle Pole Organization
Source: Front Plant Sci. 2016 Aug 25;7:1277. doi: 10.3389/fpls.2016.01277 (PMC4997046; doi:10.3389/fpls.2016.01277)

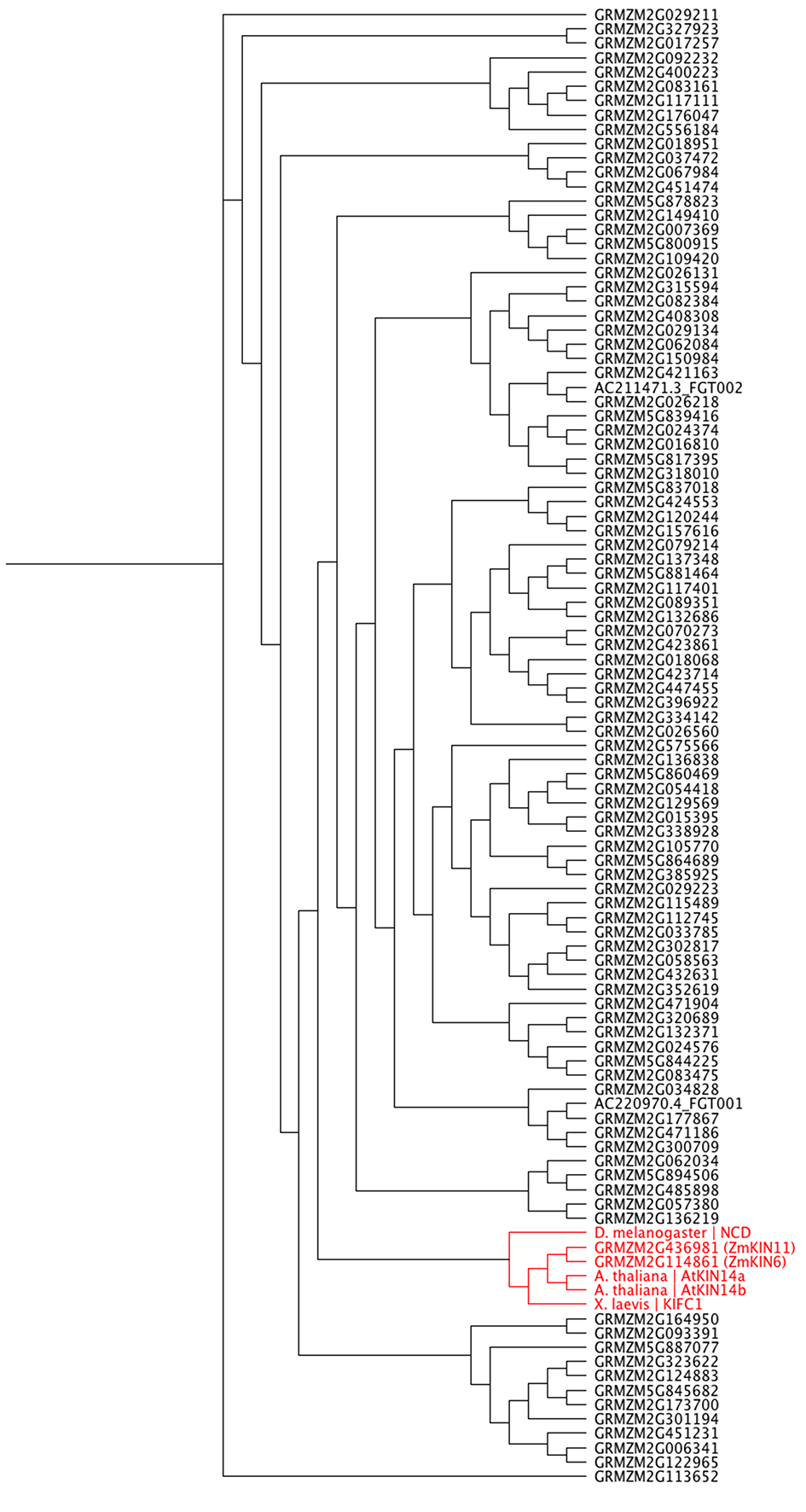

Supplement: Figure S1 — Alignment of maize kinesins reveals two members of the kinesin-14A subfamily. Coding sequences were aligned using the MUSCLE algorithm. All genes shown are from the Zea mays B73 reference genome unless a different species name is given. Two maize genes appear with other members of the kinesin-14A subfamily (highlighted in red) and were identified as candidates for dv1. The figure was generated using the software Geneious (v8.0). [file Image1.TIF]

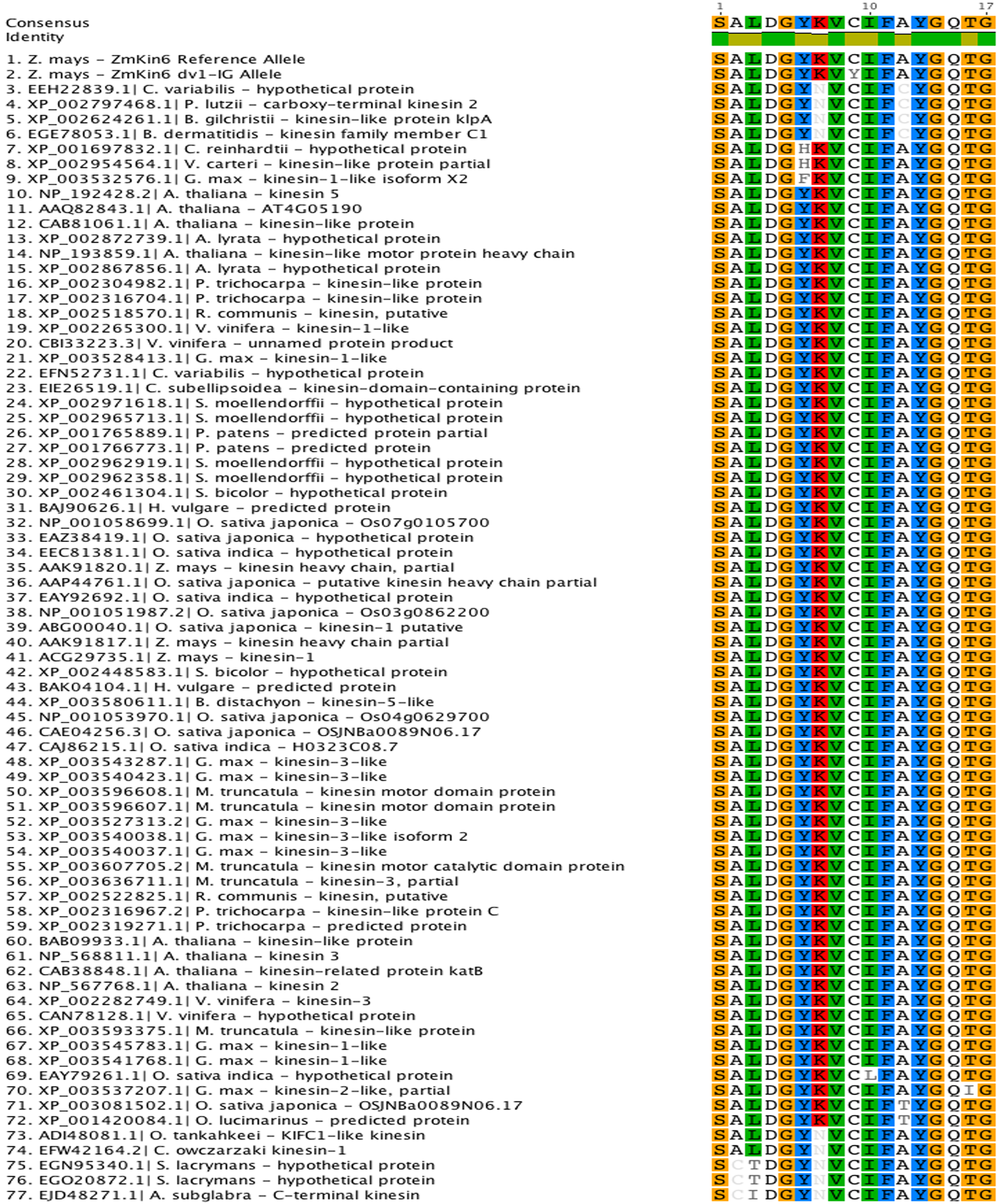

Supplement: Figure S2 — PROVEAN output indicates the residue at which the dv1-IG mutation occurs is highly conserved. The PROVEAN software identified 75 GenBank entries that show homology to the Dv1 sequence. GenBank IDs are listed for each entry along with their species and given annotation. A portion of the PROVEAN alignment is shown at the right. Amino acid sequences for the B73 reference and dv1-IG allele are listed at the top. This cysteine in the middle of the alignment (residue 494) is conserved across the sample of plant, algae, and animal sequences. However, the dv1-IG allele codes for a tyrosine at this position. The figure was generated using the software Geneious (v8.0). [file Image2.TIF]
